# Comparison of differentiated thyroid carcinoma recurrence and its clinical features in children of different ages

**DOI:** 10.18632/oncotarget.18229

**Published:** 2017-05-26

**Authors:** Bin Ye, Jun Shi, Chenling Shen, Longhao Wang, Haixia Hu, Yan Ma, Quan Wang, Jingrong Lu, Guangjun Yu, Mingliang Xiang

**Affiliations:** ^1^ Department of Otolaryngology Head and Neck Surgery, Xinhua Hospital, Shanghai Jiao Tong University School of Medicine, Shanghai, China; ^2^ Shanghai Jiao Tong University School of Medicine, Shanghai, China

**Keywords:** differentiated thyroid carcinoma, recurrence, recurrence-free survival, pediatrics

## Abstract

The prevalence of differentiated thyroid carcinoma (DTC) in children is increasing. However, the clinical features and recurrence of DTC in children in different age groups, especially those less than 14 years old, are not well studied. We retrospectively investigated 73 children diagnosed with DTC in our hospital between January 1998 and July 2014. Data were reviewed for different age groups based on the age at initial diagnosis: 5-9, 10-14, or 15-19 years. The mean age of the recurrence group (10.6±4.1 years) was lower than that of the non-recurrence group (12.6±6.2 years; *P*=0.004). The main symptom at initial diagnosis was local invasion in the recurrence group, but was thyroid nodules in the non-recurrence group (*P*<0.001). The recurrence and non-recurrence groups did not differ in TNM stage or risk level. However, according to our age classification, the American Thyroid Association pediatric risk level was significantly different in three age groups (P=0.024). The DTC recurrence rate in each age group decreased as the age of the children increased (*P*=0.011). Thus, a high risk of recurrence and a high proportion of local invasion cases were observed in the youngest age group, suggesting that younger age is an important risk factor for DTC recurrence in children.

## INTRODUCTION

The proportion of newly diagnosed differentiated thyroid carcinoma (DTC) cases is increasing among individuals under the age of 20 [1–3]. The symptoms of DTC in children are not usually typical upon initial diagnosis; while DTC may present as a neck mass (with or without a thyroid lesion) in some children, in others, DTC may not even be diagnosed until the discovery of distant metastases. Children with DTC have a high degree of extrathyroid extension and high rates of distant metastasis and recurrence [4–6]; nonetheless, the prognosis for these patients is usually good, and disease-specific death rarely occurs [7–9].

A more aggressive presentation and higher distant metastasis rate have been observed in pre-pubertal children than in adolescents with DTC [5]. However, previous studies of DTC have included considerable proportions of adolescents [1, 10–13]. The clinical features of children with DTC, especially children less than 10 years old, are still not well understood. Indeed, according to 2015 guidelines, the specific circumstances of each age group of DTC patients have not yet been explained in detail, especially for younger age groups [14].

Thus, in the present study, 73 children with DTC treated in our hospital were retrospectively evaluated by age group. The same diagnostic and therapeutic techniques were employed for all subjects. We compared the age groups in terms of their clinical presentation, TNM stage, risk level of recurrence, and other characteristics.

## RESULTS

In total, 73 children with DTC were included: 54 cases without recurrence and 19 cases with recurrence. The mean age of the recurrence group was 10.6±4.1 years, which was lower than that of the non-recurrence group (12.6±6.2 years; *P*=0.004). The main symptom in the recurrence group at initial diagnosis was local invasion (13 cases, 68.4%) (*P*<0.001), while the main initial symptom in the non-recurrence group was thyroid nodules (35 cases, 65.0%) (*P*<0.001).

The gender proportions of the two groups were similar, with both groups having slightly more females than males. Significant differences were not observed in tumor size, thyroid nodule number and cervical lymph node metastasis between the recurrence and non-recurrence groups (Table 1). When Tumor-Node-Metastasis (TNM) staging was performed according to the 2015 American Joint Committee on Cancer guidelines, the two groups did not differ significantly in TNM stage. The numbers (percentages) of T1a, T1b, T2, and N0 tumors in the recurrence group were 1 (10.5%), 4 (21.1%), 8 (42.1%), and 11 (57.9%), respectively, while the numbers (percentages) in the non-recurrence group were 9 (16.7%), 9 (16.7%), 23 (42.6%), and 23 (42.6%), respectively. The risk levels also did not differ significantly between the recurrence and non-recurrence groups. In the recurrence group, there were 8 (42.1%), 3 (15.8%) and 8 (42.1%) low-, intermediate- and high-risk cases, respectively, and in the non-recurrence group, there were 21 (38.9%), 15 (27.8%), and 18 (33.3%) low-, intermediate- and high-risk cases, respectively (Table 1).

**Table 1:** Characteristics of children in the recurrence and non-recurrence groups

Characteristic	Non-recurrence group	Recurrence group	P
	n=54	n=19	
**Age at diagnosis (years, mean±SD)**	12.6±6.24	10.6±4.1	0.004
**Sex (male:female)**	17:37 (31.5% male)	6:13 (31.6% male)	NS
**Status at diagnosis**			
Thyroid nodules	35 (65.0%)	7 (36.8%)	0.0003
Cervical mass	23 (42.6%)	14 (73.7%)	0.835
Local invasion	1 (1.9%)	13 (68.4%)	9×10^-6^
**Primary tumor**			
Mean size (cm, mean±SD)	2.2±1.2	2.4±1.4	NS
Unilateral thyroid nodule	37 (68.5%)	15 (78.9%)	
Multifocality	16 (29.6%)	3 (15.8%)	
**LN metastases**	26 (48.1%)	5 (26.3%)	NS
Ipsilateral	18 (33.3%)	5 (26.3%)	
Bilateral	8 (14.8%)	0	
**Lung metastases**	4 (7.4%)	1 (5.3%)	NS
**Tumor stage**			
T1a	9 (16.7%)	1 (10.5%)	NS
T1b	9 (16.7%)	4 (21.1%)	
T2	23 (42.6%)	8 (42.1%)	
T3	4 (5.55%)	2 (10.5%)	
T4a	7 (13.0%)	4 (21.1%)	
T4b	2 (3.7%)	0	
**N stage**			
N0	23 (42.6%)	11 (57.9%)	NS
N1a	16 (29.6%)	3 (15.8%)	
N1b	15 (27.8%)	5 (26.3%)	
**M stage**			
M0	50 (92.6%)	17 (89.5%)	NS
M1	4 (7.4%)	2 (10.5%)	
**ATA pediatric risk level**			
Low	21 (38.9%)	8 (42.1%)	NS
Intermediate	15 (27.8%)	3 (15.8%)	
High	18 (33.3%)	8 (42.1%)	
**Surgical procedure**			
Unilateral lobectomy	7 (13.0%)	2 (10.5%)	NS
Unilateral lobectomy with IV region dissection	14 (25.9%)	6 (31.6%)	
TT with IV region dissection	33 (61.1%)	11 (57.9%)	
**Lateral cervical lymph node dissection**			NS
Ipsilateral	18 (33.3%)	6 (31.6%)	
Bilateral	7 (13.0%)	2 (10.5%)	
Tracheotomy and other procedure	1 (1.9%)	0	
**Histology**			NS
Papillary	39 (72.2%)	16(84.2%)	
Papillary with follicular variant	8 (14.8%)	1(5.3%)	
Follicular	6 (11.1%)	0	
Medullary	1 (1.9%)	2(10.5%)	
**Postoperative complications**			NS
Recurrent laryngeal nerve damage	4 (7.4%)	1 (5.3%)	
Hypocalcaemia	3 (5.55%)	2 (10.5%)	
**Postoperative radioiodine treatment**	39 (72.2%)	11 (57.9%)	NS
**LT-4 replacement**	45 (83.3%)	13 (68.4%)	NS
**Follow-up status**			
Distant or regional metastasis after treatment	8 (14.8%)	6 (31.6%)	NS

NS: not significant; LN: lymph nodes; TT: total thyroidectomy. The TNM classification system and ATA pediatric risk levels were based on the Guidelines for Children with Thyroid Nodules and Differentiated Thyroid Cancer [14].

Differences were not observed in operative procedures, postoperative complications or pathology between the two groups (Table 1). Pathological classifications of papillary, papillary with follicular variant, follicular, and medullary carcinoma did not differ significantly between the two groups (Table 1). The frequency of radioiodine (I^131^) and L-thyroxine treatment was slightly lower in the recurrence group than in the non-recurrence group, though the difference was not significant. In addition, the frequency of lung metastasis in the recurrence group was not significantly different from that in the non-recurrence group (Table 1).

Next, the 73 children with DTC were divided into three groups with five-year age intervals, according to their age at diagnosis. Among them, 21 patients were 5-9 years old, 16 patients were 10-14 years old, and 36 patients were 15-19 years old. The follow-up times for the age groups were 70.6±27.6, 59.3±37.4, and 53.0±30.9 months, respectively (Table 2).

**Table 2:** Differences in TNM staging and risk level among the age groups of children with DTC

Characteristic	Age at diagnosis (years)			
	5-9	10-14	15-19	P
	n=21	n=16	n=36	
**Age at diagnosis (years, mean±SD)**	7.5±1.4	11.8±1.6	16.9±1.4	0.000
**Sex (male:female)**	12:9 (57.1% male)	5:11 (31.3% male)	6:30 (16.7% male)	0.007
**Status at diagnosis**				
Thyroid nodules	9 (42.9%)	9 (56.3%)	24 (68.8%)	0.044
Cervical mass	14 (66.7%)	11 (68.8%)	12 (33.3%)	0.161
Local invasion	8 (38.1%)	1 (6.3%)	5 (13.9%)	0.091
**Primary tumor**				
Mean size (cm, mean±SD)	2.4±1.4	2.4±0.7	2.1±1.3	NS
**Histology**				NS
Papillary	16 (76.2%)	13 (81.2%)	26 (72.2%)	
Papillary with follicular variant	2 (9.5%)	2 (12.5%)	5 (13.9%)	
Follicular	2 (9.5%)	0	4 (11.1%)	
Medullary	1 (4.8%)	1 (6.25%)	1 (2.8%)	
**Tumor stage**				NS
T1a	1 (4.8%)	1 (6.3%)	9 (25%)	
T1b	4 (19.0%)	3 (18.8%)	6 (16.7%)	
T2	8 (38.1%)	11 (68.8%)	12 (33.3%)	
T3	1 (4.8%)	1 (6.3%)	3 (8.3%)	
T4a	6 (28.6%)	0	5 (13.9%)	
T4b	1 (4.8%)	0	1 (2.8%)	
**N stage**				NS
N0	9 (42.6%)	7 (43.8%)	18 (50.0%)	
N1a	4 (19.0%)	4 (25.0%)	11 (30.6%)	
N1b	8 (38.1%)	5 (31.2%)	7 (19.4%)	
**M stage**				NS
M0	19 (90.5%)	13 (81.3%)	35 (97.2%)	
M1	2 (9.5%)	3 (18.8%)	1 (2.8%)	
**ATA pediatric risk level**				0.024
Low	6 (28.6%)	6 (37.5%)	17 (47.2%)	0.011*
Intermediate	2 (9.5%)	4 (25.0%)	12 (33.3%)	
High	13 (61.9%)	6 (37.5%)	7 (19.4%)	
**Surgical procedure**				NS
Unilateral lobectomy	3 (14.3%)	2 (12.5%)	4 (11.1%)	
Unilateral lobectomy with IV region dissection	3 (14.3%)	5 (31.3%)	12 (33.3%)	
TT with IV region dissection	15 (71.4%)	9 (56.3%)	20 (27.4%)	
**Lateral cervical lymph node dissection**				NS
Ipsilateral	7 (33.3%)	5 (31.3%)	12 (33.3%)	
Bilateral	3 (4.3%)	3 (18.8%)	3 (8.3%)	
**Tracheotomy and other procedure**	1 (4.8%)	0	0	NS
**Postoperative complications**				NS
Recurrent laryngeal nerve damage	2 (9.5%)	1 (6.3%)	2 (5.5%)	
Hypocalcaemia	2 (9.5%)	1 (6.3%)	2 (5.5%)	
**Postoperative radioiodine treatment**	11 (52.4%)	9 (56.3%)	24 (66.7%)	NS
**LT-4 replacement**	17 (80.1%)	13 (81.3%)	25 (69.4%)	NS
**Median follow-up, months (range)**	62 (36-120)	45 (18-126)	48 (17-132)	
**Follow-up status**				
Distant or regional metastasis after treatment	5 (23.8%)	5 (31.3%)	4 (11.1%)	NS
**Recurrence**	9 (42.9%)	5 (31.3%)	5 (13.9%)	0.046

NS: not significant; LN: lymph nodes; TT: total thyroidectomy. The TNM classification system and ATA pediatric risk levels were based on the Guidelines for Children with Thyroid Nodules and Differentiated Thyroid Cancer [14].

We then determined the recurrence intervals for all the age groups, and found that the average recurrence times for the 5-9-, 10-14- and 15-19-year-old groups were 31±33.11, 31.4±19.46 and 25±16.85 months, respectively. The number of recurrent cases was 9 (42.9%) in the 5-9-year-old group, 5 (31.3%) in the 10-14-year-old group, and 5 (13.9%) in the 15-19-year-old group. The recurrence rates differed significantly among the age groups (*P*=0.046) (Table 2).

The ratios of males to females in the three groups were 12:9, 5:11, and 6:30, respectively, revealing that the proportion of males was highest in the youngest group. Initial thyroid nodule symptoms were more common in the oldest group of children with DTC (*P*=0.044), while in the younger age groups (5-9 years and 10-14 years), cervical masses were often found during routine physical examinations. The 5-9-year-old children with DTC often presented with local invasion (8 cases, 38.1%), including that of the muscle, recurrent laryngeal nerve and trachea, although the local invasion rate did not differ significantly among the groups. The tumor sizes and pathological types were similar across the age groups.

Although statistically significant differences were not observed for T, N and M stages among the age groups, the proportion of T4a-stage cases was higher in the 5-9-year-old group than in the other age groups. The number of high-risk patients was significantly higher in the 5-9-year-old group than in the other two groups (P<0.05), and the proportion of high-risk patients in each age group decreased with age (Table 2).

**Figure 1:**
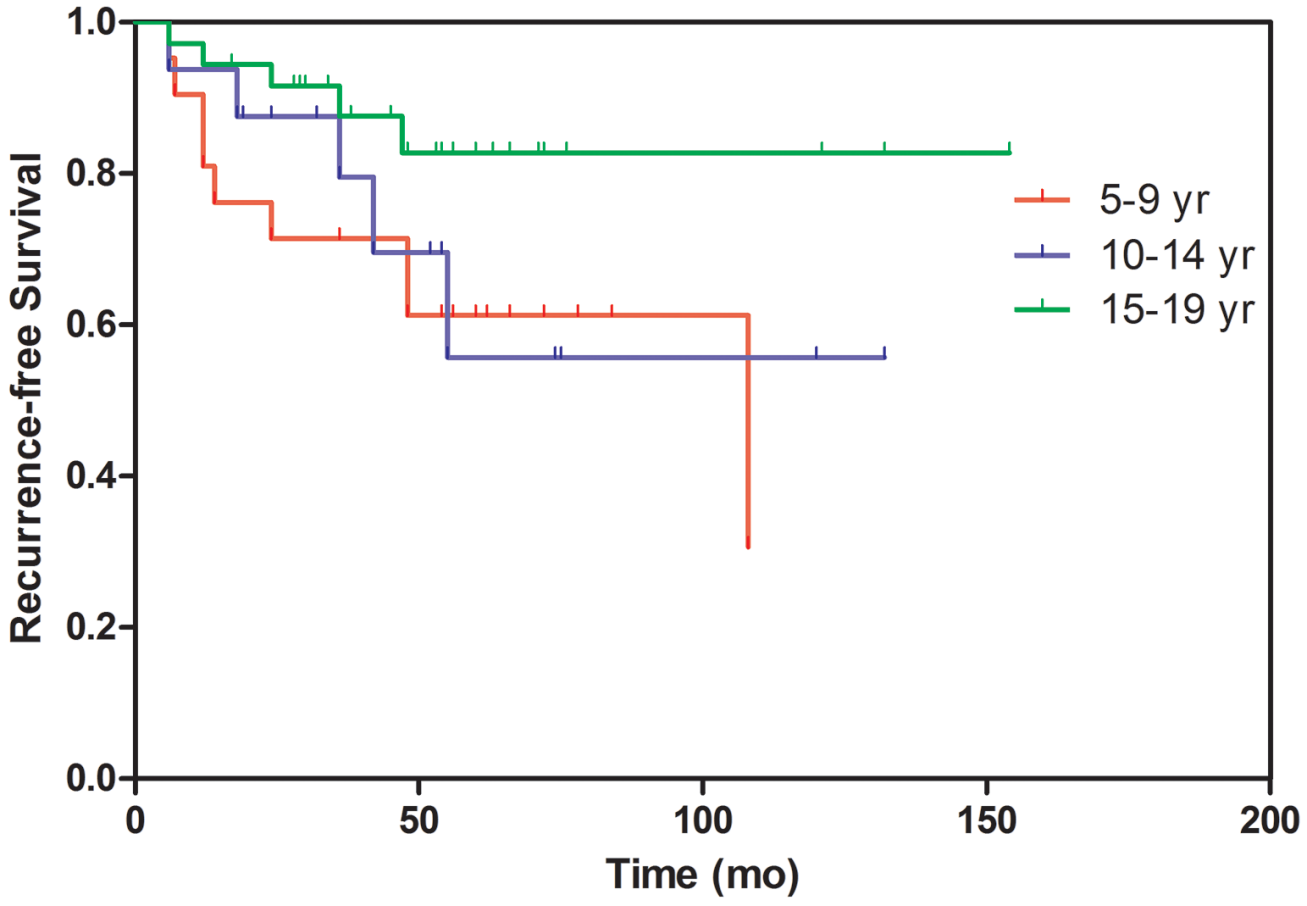
Comparison of the recurrence-free survival times among the age groups Recurrence-free survival was evaluated by the Kaplan-Meier method. The average recurrence times of the 5-9-, 10-14- and 15-19-year-old age groups were 31±33.11, 31.4±19.46 and 25±16.85 months, respectively. The recurrence-free survival times did not differ significantly among the three groups (X^2^=4.064, P=0.131).

The age groups did not differ significantly in terms of surgical procedure, postoperative complications, postoperative I^131^ proportion, L-thyroxine therapy, or lung metastasis. The recurrence-free survival times of the three groups did not differ significantly (Figure 1). During the follow-up period, no deaths were observed after surgery and I^131^ therapy, though cases of lung metastasis were recorded.

## DISCUSSION

Differences in tumor manifestation, neck recurrence and lung metastasis have been established between children and adults with DTC [15, 16], and differences have also been reported between pre-pubertal children and adolescents with DTC [5, 17]. Considering the increasing incidence of DTC in children in recent years, we analyzed the clinical features of children who had experienced DTC recurrence, and focused on the trends in age groups of five-year intervals.

The children in the non-recurrence group were older than those in the recurrence group (mean ages: 12.6±6.2 and 10.6±4.1 years, respectively), Moreover, the frequency of recurrence varied among the age groups, being 42.9% in the 5-9-year-old group, 31.3% in the 10-14-year-old group, and 13.9% in the 15-19-year-old group. This observation suggests that those initially diagnosed at a younger age may be more prone to tumor recurrence. Therefore, regular follow-ups and examinations should be emphasized for younger children with DTC [18].

When we performed TNM staging and risk stratification according to the 2015 American Joint Committee on Cancer recommendations [19], we did not observe significant differences between the recurrence and non-recurrence groups in TNM stage or risk level. The total percentage of T1a, T1b, and T2 tumors was greater than 50%, and the percentage of N0 tumors was close to 50% in non-recurrence group. The low-risk level accounted for nearly 40% of children in both the recurrence and non-recurrence groups (Table 1), which suggests that nearly half of the recurrent cases were low-risk when they were initially diagnosed. Thus, in the present study, the ATA-recommended risk factors examined during the initial diagnosis did not appear to be sufficient to predict the risk of recurrence. Further research should focus on identifying new strategies for assessing recurrence risk.

When we studied the clinical characteristics of children with DTC grouped at five-year intervals, we found that the proportion of males decreased with increasing age, whereas the proportion of females increased. Interestingly, the recurrence rate of each group decreased with increasing age. The proportions of males and females in the three groups were 12:9, 5:11, and 6:30 (Table 2), but the recurrence rates were 42.9%, 31.3%, and 13.9% (Table 2). This trend suggests that the DTC recurrence rate was related to the proportion of males.

In the 15-19-year-old adolescent group, the majority of patients were diagnosed with thyroid nodules (24 cases, 68.8%), whereas in younger children with DTC (5-9- and 10-14-year-olds), the majority of patients were diagnosed with cervical masses (66.7% and 68.8%, respectively). The rate of local invasion of the striated muscle and recurrent laryngeal nerve was higher in the 5-9-year-old group than in the other age groups, although the difference was not significant (p=0.091); thus, the proportion of high-risk cases was greater in this group (*P*=0.024) (Table 2). Such age-related clinical manifestations may be due to age-specific metabolism and genetic factors in younger children with DTC, and may contribute to the lack of complaints and delayed findings in younger children [4, 20]. These findings suggest that more aggressive tumors occur in younger children [21, 22]. Thus, we conclude that a younger age is an important risk factor for DTC recurrence in children [5, 23, 24].

Recurrence was not observed for the recurrent cases after reoperation to remove the residual thyroid and/or neck recurrences. For preoperative and postoperative pulmonary metastases, lung metastatic lesions were well-controlled after surgical resection of the lesions and isotope therapy. Although the incidences of local invasion and recurrence were higher in younger children than in older children with DTC, the survival rate was 100% after our surgical strategy and combined I^131^ and L-thyroxine treatment in all age groups. During the follow-up period, we did not observe adverse effects of these treatments on the growth and development of these children. This finding was consistent with previously reported results that, despite the serious clinical manifestations of DTC in children, the survival rate in the follow-up period was satisfactory after surgery and I^131^ and L-thyroxine treatment [11, 25, 26, 27].

In conclusion, younger children with DTC and those with local invasion as an initial symptom were prone to tumor recurrence. Nearly half of the recurrent cases were low-risk when they were initially diagnosed; therefore, follow-up investigations for low-risk children are still necessary. A high proportion of local invasion cases and a high risk of recurrence were observed in the youngest age group; thus, local invasion and younger age represent important risk factors for DTC recurrence in children. Appropriate treatment strategies should be adopted to increase the awareness and follow-up analysis of the risk of DTC in young children.

## PATIENTS AND METHODS

This paper presents a retrospective study of 73 children diagnosed with DTC in our hospital between January 1998 and July 2014. These children were diagnosed and treated by a multidisciplinary team (pediatric otolaryngology head and neck surgeons, pediatric endocrinologists, and nuclear medicine physicians). Data were collected from all patients, including the initial symptoms, TNM stage, risk level, therapy, course, and follow-up results. According to their age at initial diagnosis, the patients were divided into three age groups: the 5-9-year-old group, 10-14-year-old group, and 15-19-year-old group.

We also collected clinical data, including the age, gender, onset of symptoms, ultrasound scans, tumor size, fine-needle aspiration, histological type, invasion of cervical soft tissues, and local and distant metastases (Table 1). TNM stages and risk levels were assessed according to the Management Guidelines for Children with Thyroid Nodules and Differentiated Thyroid Cancer, as suggested by the American Thyroid Association (ATA) Guidelines Task Force on Pediatric Thyroid Cancer in 2015 [14] (Table 2).

After ultrasound or fine-needle aspiration, the patients underwent unilateral or bilateral primary surgical resection according to the location of the thyroid lesion. For unilateral DTC, the thyroid gland was unilaterally resected and the ipsilateral level VI lymph node was dissected. Bilateral lobectomy resection was performed for any patient who presented with lymph node metastasis or a local invasion of the capsule, muscle layer, nerve or trachea. For bilateral DTC, bilateral lobectomy and bilateral level VI lymph node dissection were performed. Lateral neck lymph node dissection was performed for neck lymph node lesions. For patients with preoperative and postoperative distant metastases, a series of treatments was considered, including surgery and combined I^131^ and L-thyroxine treatment.

For ultrasound- or fine-needle-aspiration-confirmed thyroid and lymph node recurrence, the appropriate operation was performed according to the range of recurrent lesions, including the removal of the residual thyroid and selective neck dissection. I^131^, thyroid-stimulating hormone and a suppressive dose of L-thyroxine were administered after reoperation.

Data were collected on postoperative complications, including hoarseness and low calcium. Postoperative I^131^ was used for residual thyroid and distant metastases, and a thyroid-stimulating-hormone-suppressive dose of L-thyroxine was used after surgery. Follow-up investigations were performed every three to six months, including repeat thyroid and cervical ultrasonography, chest X-ray examination and thyroid function tests. For patients appearing to be in remission who presented negative diagnostic whole-body scans, chest X-rays and ultrasounds, and low or normal ranges of serologic thyroglobulin, the repeated inspection interval was extended to every two years.

Disease-free remission was defined by two consecutive negative whole-body scans and ultrasounds and a normal thyroglobulin range. Cases were considered recurrent when evidence of disease was detected in ultrasound scans, thyroglobulin levels were positive, and/or whole-body scan results were positive.

Postoperative I^131^ treatment was performed according to the protocols of the Society of Nuclear Medicine and the European Thyroid Cancer Taskforce. The dose was determined from the tumor manifestation, as follows: 30-100 mCi for thyroid capsule invasion, 150 mCi for cervical lymph node metastases, and 175-200 mCi for distant metastases. The dose of I^131^ therapy was adjusted according to the child's weight. The simple estimate was calculated as follows: four-fifths of the dose was used for children aged 15-19 years, two-thirds of the dose was used for children aged 10-14 years, and one-third of the dose was used for children aged 5-9 years.

### Statistical methods

The data were statistically analyzed with SPSS software (version. 19.0, SPSS, Chicago, Illinois, USA). All data in each group were analyzed for normality with the Shapiro-Wilk test. The ages and tumor diameters of the recurrence and non-recurrence groups were compared by the Mann-Whitney test. The other parameters were compared between the two groups with a chi-square test. After the patients were divided according to five-year age intervals, the ages among the three groups were statistically compared by one-way analysis of variance followed by Fisher's Least Significant Difference test, while tumor size was analyzed by the Kruskal-Wallis test, and other parameters were analyzed with the chi-square test. Spearman correlation coefficient tests were used to determine the correlation between the ATA pediatric risk levels and age groups. The recurrence-free survival for each age group was statistically evaluated by the Kaplan-Meier method. The significance level was set at *P*<0.05.

## Abbreviations

ATA: American Thyroid Association
DTC: differentiated thyroid carcinoma
TNM: tumor-node-metastasis
